# Mechanisms of resveratrol against diabetic wound by network pharmacology and experimental validation

**DOI:** 10.1080/07853890.2023.2280811

**Published:** 2023-11-15

**Authors:** Ding Youjun, Yumeng Huang, Yongxian Lai, Zhouji Ma, Xin Wang, Bin Chen, Xiaofeng Ding, Qian Tan

**Affiliations:** a Nanjing Drum Tower Hospital, Clinical College of Jiangsu University, Nanjing, China; b Department of Emergency Surgery, The Fourth Affiliated Hospital of Jiangsu University (Zhenjiang Fourth People’s Hospital), Zhenjiang, China; c Shanghai Skin Disease Hospital, Tongji University School of Medicine, Shanghai, China; d Nanjing Drum Tower Hospital, Clinical College of Nanjing Medical University, Nanjing, China; e Jintan Affiliated Hospital of Jiangsu University, Changzhou, China; f Institute of Plant Resources and Chemistry, Nanjing Research Institute for Comprehensive Utilization of Wild Plants, Nanjing, China; g Nanjing Drum Tower Hospital, The Affiliated Hospital of Nanjing University Medical School, Nanjing, China

**Keywords:** Resveratrol, diabetic wound, inflammation, AGE-RAGE signalling pathway

## Abstract

**Background:**

Resveratrol (RSV) that possesses anti-oxidative, anti-inflammatory, and pro-angiogenic effects is an effective drug for diabetic wound (DW), while its pharmacological mechanism remains to be elucidated. In this study, we apply network pharmacology and experimental validation approach to reveal the potential mechanism of RSV against DW.

**Methods:**

We obtained potential targets for RSV and DW from the publicly available database. Using interaction networks and conducting GO and KEGG pathway enrichment analyses, we constructed target-pathway networks to explore the relationship between RSV and DW. To validate the pharmacological mechanism of RSV, we induced the DW model.

**Results:**

Ninety overlapped targets between RSV and DW were obtained, and the hub genes of the PPI network included *TNF*, *IL-6*, *CASP3*, *MAPK3*, *VEGFA*, *IL-1β*, *AKT1*, and *JUN*. Based on target-pathway networks, the AGE-RAGE signalling pathway was involved in the RSV treatment of DW. Furthermore, *in vivo* experiments revealed that RSV significantly promoted wound healing in diabetic mice and attenuated the expression of pro-inflammatory cytokines in wound tissue. Meanwhile, RSV could inhibit the AGE-RAGE signalling pathway and thus reduce the activation of NF-κB.

**Conclusion:**

This study initially revealed the biological mechanism of RSV for treating DW through multi-target and multi-pathway. AGE-RAGE, FoxO, MAPK, PI3K-AKT and other signalling pathways may be the main pathways of RSV in treating DW. RSV reduces the inflammatory response by inhibiting the AGE-RAGE signalling pathway, which in turn promotes DW healing.

## 1. Introduction

As a complex metabolic disease, diabetes mellitus was increasing prevalence, and the number is projected to rise to 640 million by 2040 [1]. Diabetes has many serious complications, and about 20% of patients suffer from diabetes wounds, which account for nearly one-third of all diabetes care costs [2]. The process of diabetic wound (DW) healing is disrupted or delayed mainly due to persistent hyperglycaemia and dyslipidemia with bacterial infection, which result in diabetic neuropathy and vasculopathy in wounds [3, 4]. Moreover, excessive inflammation with immune cell infiltration disrupts the specific stages of normal wound healing, exacerbating the difficulty of diabetes wound treatment [5, 6]. Conventional therapeutic methods such as gauze and bandages are responsible for protecting wound and absorbing tissue exudate, but they have limited functions that cannot halt disease progression [7]. Pharmacotherapy is essential for the treatment of diabetes wounds, including anti-glycemic drugs, antibiotics and antimicrobial drugs [8]. Given the bacterial resistance of antibiotics, slow wound healing and local ischaemic necrosis [9], there is an urgent need for novel drug therapies for diabetes wounds through the potential actional mechanisms.

Resveratrol (RSV) is a well-known natural polyphenol that possesses anti-oxidative, anti-inflammatory and pro-angiogenic effects, which may be a promising supportive drug for diabetes wounds. Meanwhile, several studies revealed that RSV could promote wound healing. RSV has been found to induce tissue regeneration and vascular regeneration in wounds by regulating vascular growth factors and inhibiting the expression of pro-inflammatory factors such as tumour necrosis factor alpha (TNF-α) [10, 11]. Pro-inflammatory factors could prolong the inflammatory phase and matrix metalloproteinases (MMP) damage cell migration in wound, which delay diabetes wound healing. Furthermore, the levels of pro-inflammatory markers including IL-1β, TNF-α and MMP-2, MMP-9 were reduced in patients after RSV treatment [12]. Recently, RSV was loaded into silica nanoparticles combined with hydrogels that reduce inflammation and oxidative stress in diabetes wounds, allowing the wound to receive proliferative repair signals to promote healing [13]. Huang et al. found that RSV exerted a positive role in DW healing by restoration of the activity of the SIRT1 and inhibition of FOXO1 expression to promote angiogenesis in DW [14].

**Figure 1.**
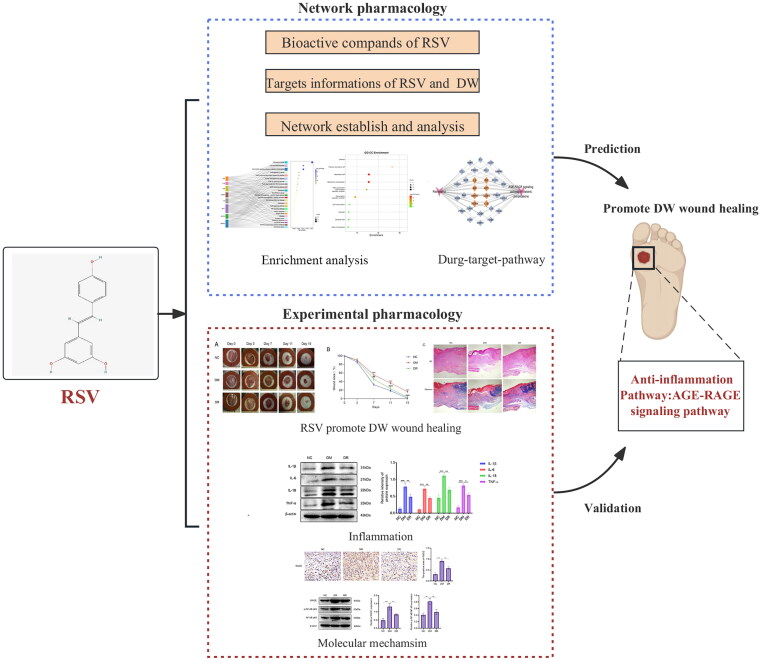
Flowchart of this experiment. RSV: resveratrol; DW: diabetic wounds.

With the quick development of bioinformatics, network pharmacology was of great importance in exploring the molecular mechanisms of traditional Chinese medicine [15]. Based on computer data analysis and network database search, network pharmacology can establish the priority of disease-related genes, and systematically predict the interaction targets and pharmacological mechanisms of herbal compounds [16, 17]. In this study, we apply network pharmacology approach to reveal the potential targets and signalling pathways of RSV against DW. Furthermore, we used the model of streptozotocin (STZ)-induced diabetic mice to further validate the pharmacological mechanism of RSV. The flow chart of this experiment is shown in Figure 1.

## 2. Methods

### 2.1. Physicochemical properties of RSV

Download the 2D structure of RSV from the PubChem database (https://pubchem.ncbi.nlm.nih.gov/) and obtain InChIKey and Isomeric SMILES . Pharmacological and molecular characterization data of RSV were obtained from the TCMSP database (http://tcmspw.com/).

### 2.2. Acquisition of potential targets for RSV and DW

Potential targets for RSV were obtained from the TCMSP database by searching for the compound name ‘resveratrol’. The databases used to obtain disease-related targets were GeneCards (https://www.genecards.org/), DisGeNET (http://www.disgenet.org/home/) and OMIM (http://www.omim.org). These three databases are publicly available, free of charge, and allow unlimited access. The search terms for diseases were ‘diabetic wounds’ and ‘diabetic foot ulcers’.

The contents of the different databases were combined, and duplicates were removed. All acquired RSV and disease targets will be standardized for gene names through the UniProt database (https://www.uniprot.org/).

### 2.3. Construction of interaction networks of intersecting genes and identification of hub genes

To find the relevant targets of RSV acting on DW, the intersection targets of both were obtained using Venn diagrams. The intersecting targets were imported into the STRING database (https://cn.string-db.org/) to construct a protein–protein interaction (PPI) network. Set the minimum required interaction score = 0.4, species = Homo sapiens. The results of the analysis were imported into Cytoscape (Version 3.9.1) software for visualization. The top 10 targets with the highest scores of the four algorithms (MNC, MCC, Degree, Closeness) were obtained using the CytoHubba plugin to identify the hub genes, and their intersection was the hub genes of the PPI network.

### 2.4. Biological pathway enrichment analysis

To explore the biological mechanisms underlying the action of RSV on DW, enrichment analysis was performed using genes that intersect RSV with DW. Kyoto Encyclopedia of Genes and Genomes (KEGG) pathway and Gene Ontology (GO) enrichment analyses were performed through the Metascape database (https://metascape.org/), restricted species = Homo sapiens, min overlap = 3, min enrichment = 1.5, *p* < .01. Based on the results of KEGG pathway enrichment analysis, 25 pathways associated with DW were displayed. GO enrichment analysis was divided into biological process (BP), cellular composition (CC) and molecular function (MF), displaying the top 10 terms of each category, respectively. The results of all biological pathway enrichment analyses were visualized using the ‘ggplot2’ (version 4.0.1) package of R software.

### 2.5. Establishing a ‘drug-target-pathway’ network

To further elucidate the specific mechanism of RSV action on DW, Cytoscape was used to visualize the KEGG pathway enrichment results and construct a ‘drug-target-pathway’ network.

The number of targets enriched in the pathway and the number of hub genes enriched in the pathway are shown.

### 2.6. Construction of the DW model and treatment

Seven-week-old male C57/B6 mice with weight of 19–23 g were provided by Jiangsu Jizui Pharmachem Biotechnology. All experimental mice were housed in a special pathogen-free condition by the Experimental Animal Centre of the First Nanjing Hospital affiliated with Nanjing Medical University. The animal experimental procedures were pre-approved by the Animal Ethics Committee of the First Nanjing Hospital (DWSY-21059103). To induce diabetic models, mice were injected intraperitoneally with STZ (50 mg/kg in 0.1 mol/L sodium citrate, pH 4.3). A model of diabetes was constructed when blood glucose level was ≥16.7 mmol/L for 30 consecutive days. Then, a full-thickness excisional wound (10 mm in diameter) was created on the upper backs of the mice. To eliminate the influence of skin contraction, a 0.6-mm thick silicone stent (inner/outer diameter = 12/20 mm) was utilized to stabilize the wound site. The stent was firmly secured to the full-thickness skin using interrupted 5–0 braided sutures and sterile gauze dressing was applied daily. All mice were divided into three groups of 10 mice each: normal control group with non-treated (NC group), diabetes model group with non-treated (DM group) and diabetes model group with 100 µL RSV (10 µmol/L; DR group). On days 0, 3, 7, 11 and 15 post-operation, the wounds were photographed and analysed by the ImageJ software.

### 2.7. Histological analysis and immunohistochemistry

Mice skin tissues were gathered on day 15, which were fixed in 4% paraformaldehyde and embedded in paraffin. Cut paraffin tissue into 5 µm-thick for haematoxylin and eosin (H&E) and Masson’s trichrome.

5 µm-thick paraffin sections were used for immunohistochemistry staining. After dewaxing, dehydration and antigen repair, the sections were soaked in 3% hydrogen peroxide for 15 min and then blocked with goat serum for 60 min. Then, the primary antibodies RAGE (WL01514, Wanleibio) were incubated with the tissue at 4 °C overnight. The next day, after washing, the sections incubate the secondary antibody at 37 °C for 30 min. Subsequently, the tissues were stained with DAB and coloured the nucleus with haematoxylin. The integrated optical density (IOD) was analysed using Image-Pro Plus 6.0 software.

### 2.8. Western blot analysis

Extract proteins from wound skin tissue using a total protein extraction kit (Solarbio, Beijing, China) and quantify protein concentration using a BCA protein assay kit (Solarbio, Beijing, China). Separate the total protein sample using SDS-PAGE gel and transfer it onto a polyvinylidene fluoride membrane. Afterwards, using TBST buffer including 5% bovine serum albumin to block the membranes and then incubated with the primary antibodies IL-1β (ab254360, Abcam), IL-6 (ab290735, Abcam), IL-18 (10663-1-AP, Proteintech), TNF-α (ab205587, Abcam), RAGE (WL01514, Wanleibio), NF-κB p65 (A19653, ABclonal), p-NF-κB p65 (3033, CST), β-actin (20536-1-AP, Proteintech) overnight at 4 °C. Then, the secondary antibody (ZB-2301) was incubated with the membranes for 1 h. Finally, the bands were detected using the ECL kit (E412-01, Vazmye), and the image was analysed using ImageJ software.

### 2.9. Statistical analysis

Statistical analysis was performed using GraphPad Prism 8 software (San Diego, CA, USA). Differences between groups were compared using one-way ANOVA, and *p* < .05 was considered a statistically significant difference.

## 3. Results

### 3.1. Information about RSV

**Figure 2.**
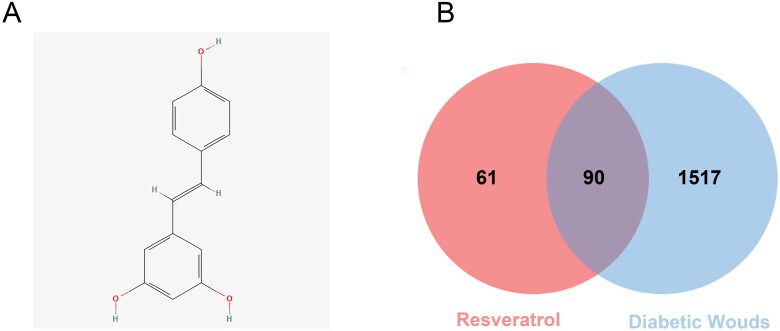
2D structure of RSV and identifying intersecting genes. (A) 2D structure of resveratrol. (B) Identification of the intersecting genes of RSV and DW. The red circles represent the action targets of RSV, the blue boxes represent DW-related targets, and the intersection of the two is the intersecting target.

The molecule ID of RSV in the TCMSP database is MOL012744. The 2D picture structure of RSV is shown in Figure 2A, and the pharmacological and molecular property data are shown in Table 1. The compound CID for RSV in the PubChem database is 445154. The Isomeric SMILES of RSV are C1 = CC(=CC = C1/C = C/C2 = CC(=CC(=C2)O)O)O. The InChIKey of RSV is LUKBXSAWLPMMSZ-OWOJBTEDSA-N.

**Table 1.** RSV-related information.

MW	Hdon	Hacc	OB (%)	Caco-2	BBB	DL	FASA-	TPSA	RBN
228.26	3	3	19.07	0.8	−0.01	0.11	0.49	60.69	2

MW: molecular weight; Hdon: hydrogen bond donor; Hacc: hydrogen bond acceptor; OB: oral bioavailability; Caco-2: Caco-2 permeability; BBB: blood–brain barrier; DL: drug-likeness; FASA: fractional negative accessible surface area; TPSA: topological polar surface area; RBN: number of rotatable bonds.

### 3.2. Target information for RSV and DW

A total of 151 potential targets for RSV were obtained from the TCMSP database, and the specific information is shown in Supplementary Table S1. All relevant targets for the disease were obtained from GeneCards, and the top 25% of the scored genes were taken according to the relevance score and merged with the targets obtained from the DisGeNET and OMIM databases. All targets were compared by the UniProt database to obtain standardized Gene Symbols. After removing all duplicate targets, a total of 1611 DW correlated targets were obtained, and the detailed information is shown in Supplementary Table S2.

### 3.3. Establishing PPI network and identifying hub genes

**Figure 3.**
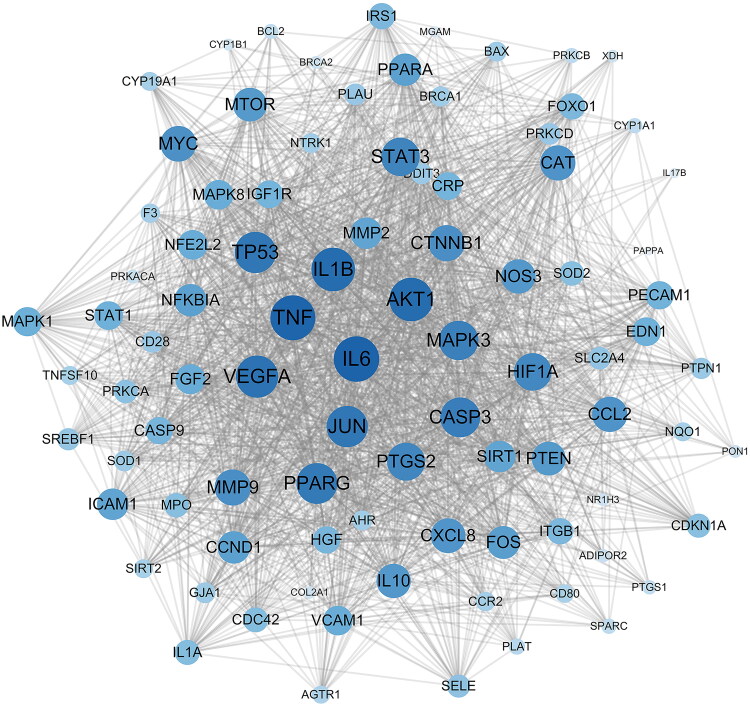
PPI network of intersecting genes. PPI network is constructed using intersecting genes, and each circle represents an intersecting gene. The darker the colour and the larger the font, the higher the degree of association with the surrounding targets. PPI: protein–protein interaction.

After obtaining the relevant targets of RSV and DW, the Venn diagram was used to screen the overlapping targets of both (Figure 2B), 90 in total, and the specific information is shown in Supplementary Table S3. The intersecting genes of RSV and DW were imported into STRING database to generate PPI network, including 90 points and 1776 edges. The results were imported into Cytoscape for visualization (Figure 3), and the scores of MNC, MCC, Degree, and Closeness were calculated using the CytoHubba plugin to filter the top 10 targets (Table 2). The intersections of the four scores were the hub genes of the PPI network: *TNF*, *IL-6*, *CASP3*, *MAPK3*, *VEGFA*, *IL-1β*, *AKT1* and *JUN*. These proteins are key targets for RSV in the treatment of DW.

**Table 2.** Hub gene score.

MCC	MNC	Degree	Closeness
TNF	TNF	TNF	TNF
STAT3	PPARG	PPARG	PPARG
MMP9	IL-6	IL-6	IL-6
IL-6	CASP3	CASP3	CASP3
CASP3	MAPK3	MAPK3	MAPK3
MAPK3	TP53	TP53	TP53
VEGFA	VEGFA	VEGFA	VEGFA
IL-1B	IL-1B	IL-1B	IL-1B
AKT1	AKT1	AKT1	AKT1
JUN	JUN	JUN	JUN

The top 10 genes for the four algorithm scores.

### 3.4. KEGG and GO enrichment analysis

**Figure 4.**
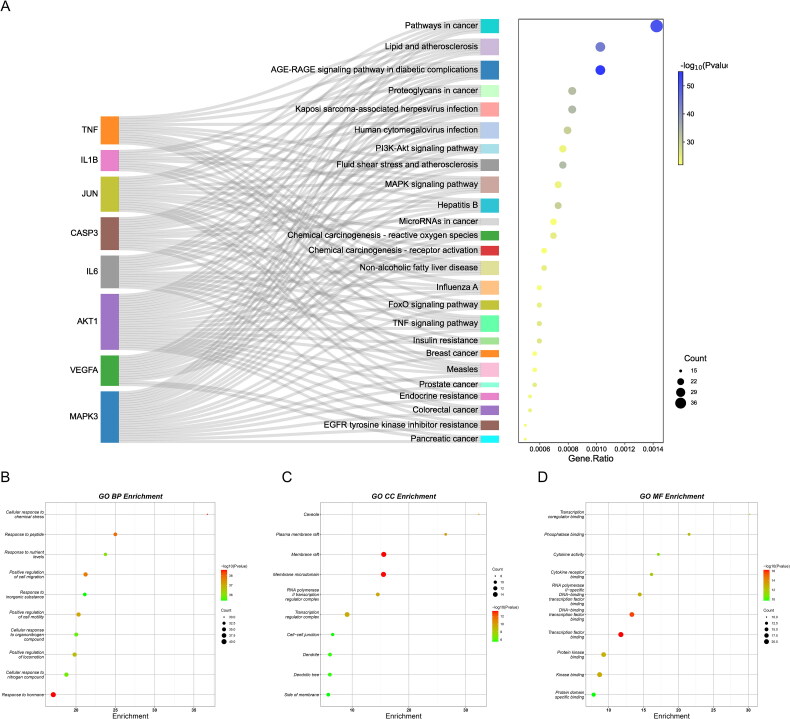
Biological pathway enrichment analysis. (A) KEGG pathway enrichment analysis. The horizontal axis indicates the enrichment analysis of the pathway. The vertical axis is the pathway name. The target on the left is the hub gene. The connecting line indicates whether the hub gene is annotated to the pathway. (B–D) GO enrichment analysis. The colour of the dot corresponds to the *p* value and the size of the dot corresponds to the number of genes annotated to the term.

**Figure 5.**
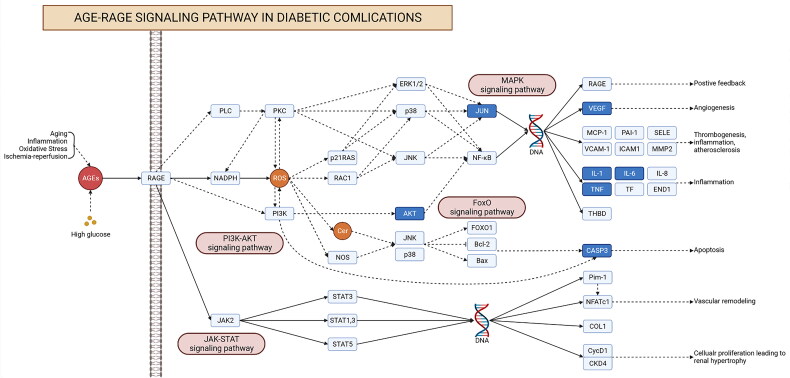
KEGG pathway diagram. Each rectangle in the diagram indicates a gene. The dark blue genes indicate intersecting genes. Realized indicates direct activation. Dashed lines indicate indirect activation.

Biological pathway enrichment analysis of 90 intersecting targets was performed through the Metascape database to explore the potential mechanisms of RSV for DW. KEGG pathway enrichment analysis identified a total of 196 pathways, and Figure 4A demonstrates 25 of them with higher association with DWs. Among them, AGE-RAGE signalling pathway, FoxO signalling pathway, MAPK signalling pathway, PI3K-Akt signalling pathway, these pathways may be closely related to RSV treatment of DW. In the GO enrichment analysis (Figure 4B–D), BP was enriched to 1682 terms, CC to 69 terms, and MF to 114 terms. The results of BP analysis identified a variety of cellular responses to substances, as well as cellular regulatory effects. The results of CC analysis were the cellular structures mainly involved in RSV treatment of DW. The results of MF analysis showed that changes in proteins, transcription factors, and cytokines may be involved in the treatment of DW by RSV. Figure 5 shows the genes in part of the pathway. Some of the intersecting genes are annotated to these pathways and there is an association between genes from different pathways.

### 3.5. Building drug-target-pathway networks

**Figure 6.**
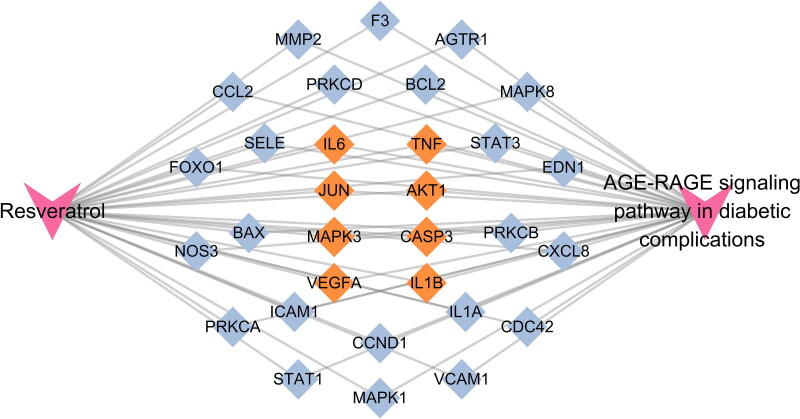
The ‘drug-target-pathway’ network diagram. Yellow targets indicate genes annotated to the pathway that are also the hub genes of the PPI network.

To further explore the potential pharmacological mechanism of RSV on DW, the ‘AGE-RAGE signalling pathway in diabetic complications’ was found to be the most closely related to RSV in the treatment of DW by combining the results of hub gene and enrichment analysis. To further observe the relationship between this pathway and related targets, a sub-network was established for demonstration (Figure 6). A total of 31 intersecting genes were found to be annotated to the pathway, including all hub genes.

### 3.6. RSV promotes wound healing in diabetic mice

**Figure 7.**
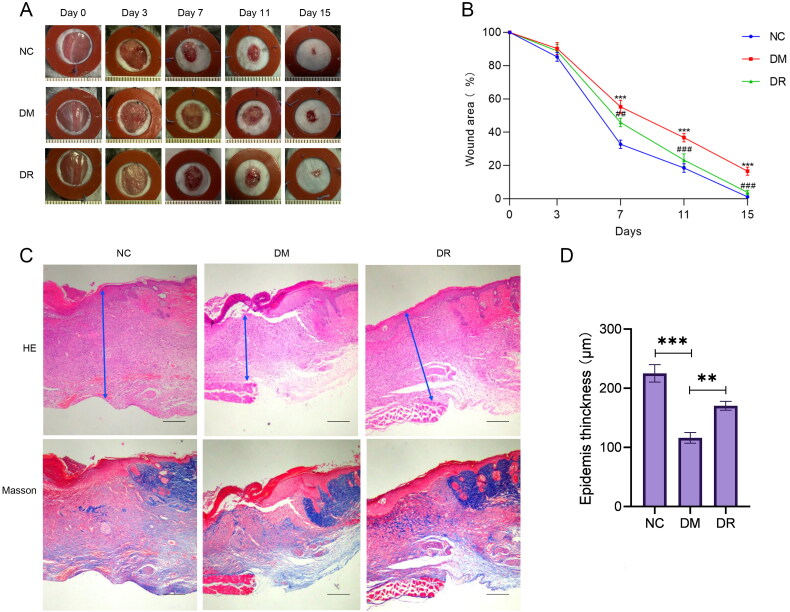
RSV promotes diabetic wound healing. (A) Pictures of rats with dorsal wounds. (B) Folding line graph of wound healing rate. (C) Histopathological pictures of wounds. The first row is H&E staining, and the second row is Masson staining (bar = 200 µm). (D) Epidermis thickness. NC: control group; DM: diabetes group; DR: diabetes + RSV group. ***p < .001 vs. NC. ^##^*p* < .01 vs. DM. ^###^*p* < .001 vs. DM.

After the wounds were successfully made on the back of the mice, the healing of the wounds was observed at different time points. As shown in Figure 7A, at day 15, the wounds of mice in the DM group were larger than those in both the NC and DR groups. From day 7 onwards, the healing rate of wounds in the DM mice group was significantly lower (*p* < .01) than that in the NC and DR groups (Figure 7B). The results of H&E and Masson staining showed that the wounds of DM mice had thinner epidermis, irregular tissue morphology, and less collagen deposition. The tissue condition of the wounds was significantly improved after the use of RSV, which was close to that of the NC group (Figure 7C,D). In conclusion, RSV can significantly promote wound healing in diabetic mice.

### 3.7. RSV improves the inflammatory state of the wound tissues

**Figure 8.**
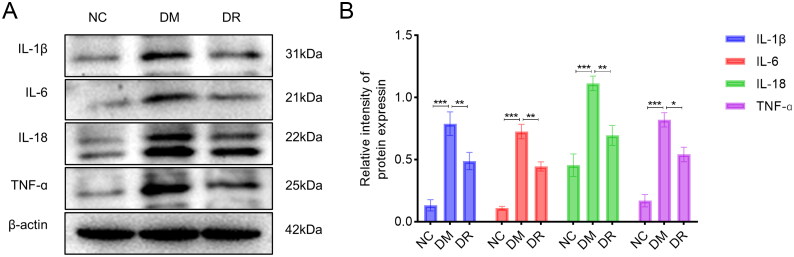
RSV reduces the expression of pro-inflammatory cytokines in DW tissues. (A) WB analysis of pro-inflammatory cytokines. (B) WB statistical plot. WB: western blot. *p < .05 vs. DM. **p < .01 vs. DM. ***p < .001 vs. DM.

To further elucidate the mechanism of action of RSV, tissues from the wound edges were collected and subjected to WB analysis. The assay results are shown in Figure 8A,B. Compared to the NC group, the expression of IL-1β, IL-6, IL-18 and TNF-α was significantly upregulated in the DM group (*p* < .001). After the use of RSV, the expression of each inflammatory cytokine was significantly down-regulated (*p* < .05 vs. DM). The above results suggest that RSV can inhibit the expression of inflammatory factors on wound tissue and improve the inflammatory status of wound tissue.

### 3.8. RSV inhibits the expression of AGE-RAGE signalling pathway

**Figure 9.**
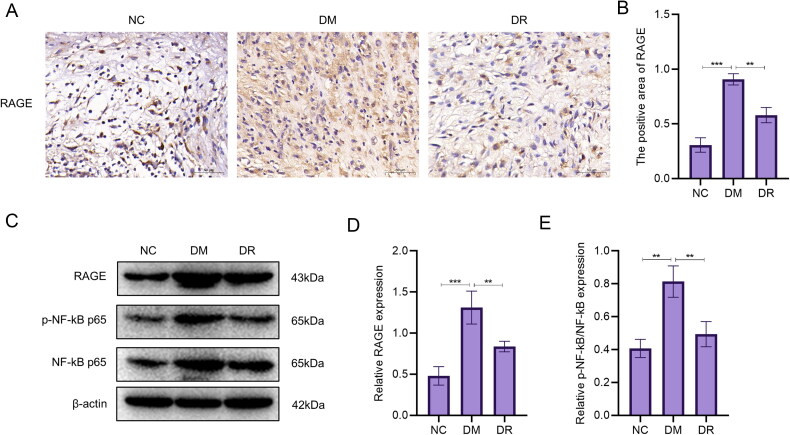
RSV inhibits the AGE-RAGE signalling pathway. (A) Immunohistochemical images of RAGE. Scale bar is 50 µm. (B) Immunohistochemical statistics of RAGE. (C) WB analysis of AGE-RAGE signalling pathway-related targets. (D) WB analysis of statistical maps.

In the results of the analysis of network pharmacology, it was found that the AGE-RAGE signalling pathway may be a key pathway in the treatment of DW by RSV. Therefore, immunohistochemical analysis of RAGE in wound tissue was performed to observe their expression. The results showed that the expression of RAGE was significantly increased in DM (*p* < .001 vs. NC; Figure 9(A,B)). After using RSV, the expression of RAGE decreased (*p* < .01 vs. DM). WB analysis showed a significant increase in RAGE expression (*p* < .001) and a significant increase in the proportion of p-NF-kB p65 (*p* < .01) in the DM group compared to the NC group (Figure 9(C,D,E)). The expression of RAGE and the percentage of p-NF-kB p65 were significantly reduced after the use of RSV (*p* < .01 vs. DM). The above results suggest that diabetes causes a significant increase in the expression of RAGE and the percentage of p-NF-kB p65, which can be reversed after the use of RSV.

## 4. Discussion

In this study, relevant targets for RSV and DW were obtained, and the overlap between the two represents a potential target for the action of RSV in treating DW. The results of the enrichment analysis showed that multiple pathways may play a role in RSV treatment of DW, among which AGE-RAGE signalling pathway in diabetic complications was significantly enriched. A PPI network was constructed using overlapping targets to identify the hub genes (*TNF*, *IL-6*, *CASP3*, *MAPK3*, *VEGFA*, *IL-1β*, *AKT1* and *JUN*) for RSV treatment of DW. These hub genes were all annotated to the AGE-RAGE signalling pathway in diabetic complications.

The main manifestation of diabetic skin ulcers is slowed wound healing, and about 20% of moderate or severe diabetic foot ulcers require amputation [18]. RSV is a naturally occurring polyphenol and plant antitoxin with anti-inflammatory, antioxidant and neuroprotective properties [19]. Several studies have revealed the role of RSV in the treatment of DW, including promotion of angiogenesis, protection of endothelial cells, and reduction of oxidative stress [20]. In addition, RSV also promoted the polarization of M2 macrophages in DW and reduced the secretion of pro-inflammatory factors [21]. Pandey et al. developed a hydrogel liposome system loaded with RSV that significantly improved ulcer healing and promoted wound tissue re-epithelialization, fibroblast proliferation, and collagen formation, while reducing inflammatory cell infiltration at the wound site [22]. Composite hydrogels containing RSV nanoparticles and platelet-derived extracellular vesicles accelerate wound healing by reducing wound inflammation to facilitate healing of the DW [13].

Wound healing is a complex process that can be broadly categorized into four distinct phases: hemostasis, inflammation, proliferation and maturation. These phases typically occur in a sequential and organized manner under normal physiological conditions [23]. The inflammatory phase is a very important one; this phase produces pro-inflammatory cytokines and promotes the clearance of pathogens and debris [23]. Early in the inflammatory phase, neutrophils are the predominant cell type within the wound tissue, and they secrete chemokines to recruit monocytes/macrophages [13, 24]. They are the dominant cell type during the inflammatory phase and play a major inflammatory regulatory role [25]. At this time, macrophages are predominantly pro-inflammatory M1 type, which secretes a variety of pro-inflammatory cytokines such as *IL-6*, *IL-1β*, *IL-12*, *TNF-α* and *iNOS* [26]. Typically, at the end of the inflammatory phase, macrophages polarize to the anti-inflammatory M2 type and contribute to tissue repair [27]. However, under the stimulation of a high glucose environment, macrophages continue to secrete pro-inflammatory cytokines such as *IL-6*, *IL-1β* and *TNF-α*, which will maintain the vicious cycle of M1 macrophage polarization and chronic inflammation [28]. By constructing a PPI network, several hub genes were identified, which played a key role in RSV treatment of DW. Among them, *IL-6*, *IL-1β* and *TNF* are inflammatory cytokines. *VEGFA*, a regulator of vascular permeability [29, 30], has been demonstrated that macrophage-specific ablation leads to delayed epithelialization, resulting in reduced expression of *VEGF* and *TGF-β1*, which is detrimental to wound angiogenesis and cell proliferation [31, 32]. In type 2 diabetes, activation of *CASP3* inhibits the expression of apoptosis protective proteins, which in turn causes apoptosis [33]. The activation of the MAPK signalling pathway can cause an inflammatory response leading to apoptosis and injury [34]. *AKT1* is a key component of the PI3K/AKT signalling pathway and regulates many BPs, including metabolism, proliferation, migration, growth, and angiogenesis [35]. Fewer studies have been conducted on the relationship between JUN and DW. Through animal experiments, we found that the expression of inflammatory cytokines was increased in DW, while the use of RSV reduced the expression of inflammatory cytokines. This suggests that inflammation affects the healing of DW, which can be ameliorated by RSV.

Combined with enrichment analysis and hub genes, the AGE-RAGE signalling pathway in diabetic complications was found to be a possible key pathway for RSV treatment of DW. Advanced glycation end products (AGEs) are formed by non-enzymatic reactions in chronic hyperglycaemic states and are toxic and immunogenic [36]. The receptor for advanced glycation end products (RAGE) is a transmembrane receptor that is widely expressed on the surface of a variety of cells, including endothelial cells, immune cells, and neurons [37]. There is clinical evidence that the levels of AGEs are significantly higher in diabetic patients than in the normal population [38]. AGE-RAGE interactions lead to tissue damage and inflammatory responses, which are the underlying mechanisms of type 2 diabetic complications [39]. It has been found that abnormal accumulation of AGEs in wound tissue increases the pro-inflammatory response of M1 macrophages while inhibiting the polarization and anti-inflammatory function of M2 macrophages, and that inhibition of the AGE-RAGE signalling pathway ameliorates macrophage dysfunction in the early stages of inflammation [40]. In addition, AGE-RAGE signalling pathway can also regulate BPs, such as gene expression, apoptosis, and oxidative stress. Under pathological diabetic conditions, the level of AGE is elevated and the expression of RAGE is upregulated, and the combination of both initiates multiple signalling pathways, including NF-κB, MAPK, and PI3K/AKT, which in turn leads to enhanced oxidative stress, increased expression of pro-inflammatory factors, and increased cellular damage [41]. NF-κB has the ability to enhance the transcription of various proteins, such as ET-1, ICAM, VCAM, TNF-α, and interleukins. This cascade response contributes to the aggravation of tissue inflammation and the generation of reactive oxygen species [42]. Additionally, the activation of NF-κB stimulates the binding of AGE to RAGE, resulting in the formation of a detrimental feedback loop [43]. It has been demonstrated that blocking pro-inflammatory RAGE/NF-κB signalling can inhibit AGE-induced TNF-α production [44]. In addition to DW, the AGE-RAGE signalling pathway plays an important role in other diabetic complications. Kim et al. found in an STZ-induced diabetic rat model that AGEs accumulated in retinal microvascular cells, accompanied by activation of NF-κB and increased expression of iNOS [45]. Activated NF-κB further leads to loss and apoptosis of pericytes in the retina [46, 47]. In diabetic nephropathy, the accumulation of AGEs in renal tissues is positively correlated with the severity of diabetic nephropathy [48]. The results of this study are consistent with those of previous studies. The expression of AGE-RAGE signalling pathway-related proteins was elevated in DW tissues. The expression of AGE and RAGE was down-regulated by using RSV, and the expression of downstream p-NF-κB p65 was reduced. It indicates that RSV can inhibit the signalling of this pathway and thus inhibit the activation of NF-κB.

This study still has some shortcomings. The main component of the study was inflammation, and no validation was performed for the other components of the results of the network pharmacology analysis. A more diverse approach could be used to reflect the therapeutic effect of RSV on DW, such as increasing *in vitro* research and deepening mechanism exploration. In the future, we can combine different drug delivery materials to further improve the therapeutic efficiency of RSV.

## 5. Conclusion

RSV has a potential multi-target and multi-pathway molecular mechanism of action in the treatment of DW. TNF, IL-6, CASP3, MAPK3, VEGFA, IL-1B, AKT1, and JUN may be the direct targets of RSV. RSV may play a role in promoting DW healing by reducing the inflammatory response and inhibiting the AGE-RAGE signalling pathway.

## Supplementary Material

Supplemental Material

## Data Availability

The data used to support the findings of this study are available by request to the corresponding author.
